# Etymologia: *Balamuthia mandrillaris*

**DOI:** 10.3201/eid2105.ET2105

**Published:** 2015-05

**Keywords:** etymologia, *Balamuthia mandrillaris*, amebae, parasites, William Balamuth, organ transplantation

## *Balamuthia mandrillaris* [balʺə-mooʹthe-ə manʺdril-aʹris]

A free-living ameba naturally found in the environment, *Balamuthia mandrillaris* can cause a serious infection of the brain, other organs (skin, liver, kidneys), and rarely, spinal cord. Originally isolated from the brain of a mandrill that died of meningoencephalitis at the San Diego Zoo, *Balamuthia mandrillaris* is named for the late professor William Balamuth of the University of California at Berkeley, for his contributions to the study of amebae. More recently, *B. mandrillaris* has been shown to be transmissible through organ transplantation.
